# Pattern of outsole shoe heel wear in infantry recruits

**DOI:** 10.1186/1757-1146-5-27

**Published:** 2012-10-25

**Authors:** Aharon S Finestone, Kaloyan Petrov, Gabriel Agar, Assaf Honig, Eran Tamir, Charles Milgrom

**Affiliations:** 1 Department of Orthopedics, Assaf Harofeh Medical Center, Zerifin, Israel; 2 Sackler Faculty of Medicine, Tel Aviv University, Ramat Aviv, Israel; 3 Israel Defense Forces Medical Corps, Tel HaShomer, Israel; 4 Department of Orthopedics, Hadassah University Hospital, Jerusalem, Israel; 5 Department of Neurology, Hadassah University Hospital, Jerusalem, Israel

**Keywords:** Durometer, Shoe wear, Shoe abrasion, Foot progression angle

## Abstract

**Background:**

Excessive shoe heel abrasion is of concern to patients, parents and shoe manufacturers, but little scientific information is available. The purpose of this study was to describe the phenomenon in a group of infantry recruits performing similar physical activity, and search for biomechanical factors that might be related.

**Methods:**

Seventy-six subjects (median age 19) enrolled. Pre-training parameters measured included height, weight, tibial length, foot arch height and foot progression angle. Digital plantar pressure maps were taken to calculate arch indexes. Shoe heel abrasion was assessed manually after 14 weeks of training with different-sized clock transparencies and a calliper.

**Results:**

Outsole abrasion was posterolateral, averaging 12 degrees on each shoe. The average heel volume that was eroded was almost 5 cm^3^. The angle of maximum wear was related to right foot progression angle (*r* = 0.27, *p* = 0.02). Recruits with lateral ankle sprains had higher angles of maximal abrasion (17° versus 10°, *p* = 0.26) and recruits with lateral heel abrasion had more lateral ankle sprains (14% versus 3%, *p* = 0.12).

**Conclusion:**

While shoe heel wear affects many people, very little has been done to measure it. In this study in healthy subjects, we found the main abrasion to be posterolateral. This seems to be related to foot progression angle. It was not related to hindfoot valgus/varus or other factors related to subtalar joint motion. These findings do not warrant modification of subtalar joint motion in order to limit shoe heel abrasion.

## Background

The issue of shoe outsole wear (abrasion as in “wear and tear”) has only been infrequently studied in the medical literature, even though patients frequently consult clinicians about the wear patterns on their shoes. While most foot clinicians have probably observed that the lateral side of the heel wears more frequently than other areas, we are not aware of any studies quantifying this phenomenon. A possible explanation for the predominantly lateral heel abrasion is the initial lateral strike of the heel when walking and running noted originally by Barnett et al. [1] and Grundy et al. [2] and later better described by Cavanagh et al. [3,4]. Lateral or medial wear of the outsole is frequently attributed to walking patterns, such as over-pronation, possibly correctable by orthotics [5,6]. One of the problems studying this field is the high variations, both in shoes people wear and in their activity. Military recruits are an ideal population to study the phenomena of outsole shoe abrasion [7]. They wear the same shoes and do the same training while under strict surveillance. Additionally, because of the high intensity of their training, outsole shoe abrasion occurs in a relatively short period of time.

The main purpose of this study was to measure the outsole shoe abrasion in a group of training military recruits. Secondary aims were to see if there are any identifiable factors in their walking pattern that can be related to the wear pattern or its magnitude, or if subjects with eroded heels are more prone to specific injuries.

## Methods

This study was an observational, prospective study on the wear pattern and its relation to overuse injuries. The study was approved by the Institutional Review Board of the Israeli Defence Forces (IDF). All participants were informed of the objectives, risks and benefits of the study and signed an informed consent.

**Figure 1:**
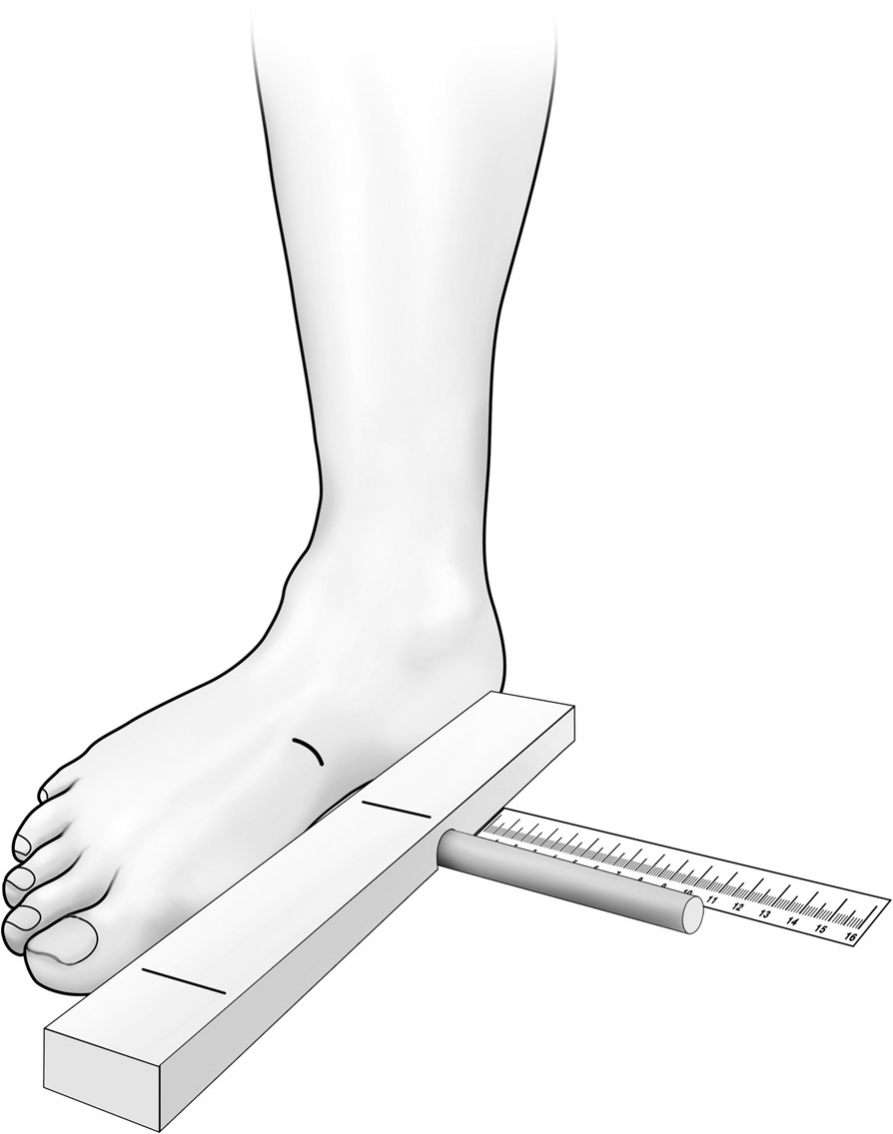
**Measuring arch height.** The navicular prominence has been marked on the foot. The examiner approximated the foot to the bar, positioning the marking on the navicular over the marking on the bar and instructed the subject to stand bearing weight, at ease. The flat ended cylindrical gauge was introduced up till skin contact, and the depth in millimetres was read off the ruler.

The recruits were examined on the military training base prior to the start of basic training. A history was taken that included date of birth, pre-induction sports participation, pre-training injuries and use of orthotics. The use of semi-rigid or rigid orthotics was an exclusion criterion, but none of the recruits had such orthotics. Prior to anthropometric measurements, an orthopaedic surgeon marked the skin above the relevant anatomical landmarks. Physical examination included measurement of height, weight and tibial length. Foot arch height was measured as the distance a flat ended circular cylinder gauge (10 mm in diameter) could be inserted under the arch immediately under the navicular prominence, till engagement with the skin. This was performed standing on a custom-made device that enabled the subject to approximate the medial aspect of the foot to a bar for standardization (Figure 1). Foot progression angle was measured by having the recruits walk barefoot over a 5 meter tiled floor covered with a thin layer of sea sand. The examiner marked the line bisecting the tangents of the feet on the third to fifth steps, measured the angle between the progression (floor tiles) and the bisection, and calculated the mean of the three steps for each side.

### Footprint analysis

**Figure 2:**
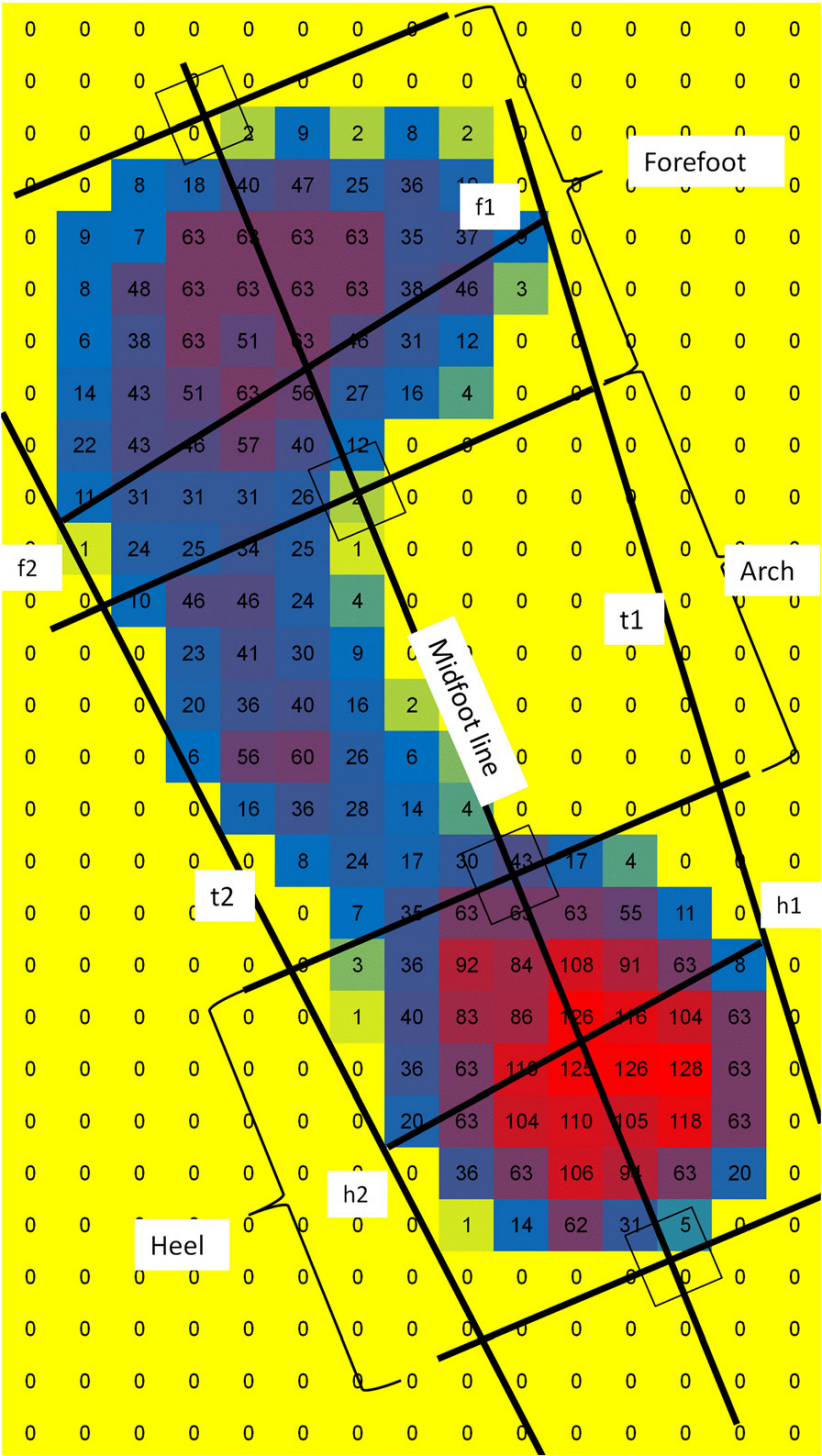
**Plantar pressure image with arch calculation.** Two tangents (t1,t2) were made by the software on either side of the foot (after removing toes), intersecting the foot perimeter at f1,f2, h1,h2. The forefoot line (f1-f2) and the heel line (h1-h2) were bisected, and the midfoot line connected the centres of these. Perpendiculars were drawn to the midfoot line at each end of the foot, and it was then divided into three equal sections, and two more perpendiculars then divided the foot into the forefoot, the arch and the heel.

Digital footprints were obtained by the subject standing at ease while facing forward on a standard commercial footprint plate with one sensor/cm^2^ (Footgraph 9800268, Belgium). The plantar pressure map, ignoring the toes, was divided into three areas as described by Chu et al. (Figure 2) [8]. Medial and lateral tangents (t1 and t2) were calculated by connecting the contact points at the heel and forefoot (f1-h1, f2-h2). The forefoot and hindfoot lines (f1-f2, h1-h2) were bisected to create the midfoot line. Four perpendicular lines were created on the midfoot line: two tangents to the ends of the pressure map, and two at the thirds of the line. The latter two divided the pressure map into three sections. The arch index (AI, area of the middle third divided by the total area) and the modified arch index (MAI, the integral of the pressure of the middle third by area divided by the integral of the total pressure) were calculated. Footprint data were also analyzed according to Brosh and Arcan because of the association they found in their analysis with stress fracture risk [9].

### Hindfoot alignment and motion measurements

The hindfoot was marked in four spots by an orthopaedic surgeon with the subject prone, feet over the end of the table. Two lower markers, representing the calcaneal segment, were placed below the axis of subtalar movement, the lowest being on the calcaneal tubercle and the other 1 cm below the axis of subtalar movement. The upper two markers, representing the leg segment, were placed 2 cm and 8 cm proximal to the axis of subtalar movement, in the midline of the Achilles tendon [10]. The subjects’ hindfeet were photographed digitally and the angles were measured by computer screen analysis of the photographs both standing normally and standing on tiptoes. Lines were drawn connecting the pairs of markings, and the angle between the lines and the horizontal going laterally gave the calcaneal and tibial angles. Tibial-calcaneal (valgus) angle was calculated by subtracting the tibial from the calcaneal angle. Hindfoot motion was calculated by subtracting the valgus on tiptoes from the valgus standing at ease.

### Outsole wear analysis

**Figure 3:**
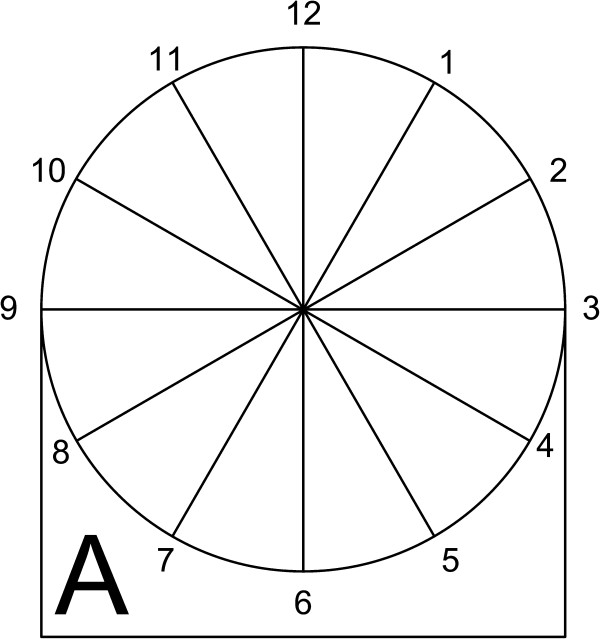
**Clock method of measuring outsole heel wear.** The clock transparency: “A” indicates that this was the smallest of the four transparencies.

The recruits trained in standard issue IDF boots for men, manufactured by Brill Shoe Industries Ltd. (Rishon le Zion, Israel) on lasts designed by the IDF. Their soles are injected with bi-layered rubber, the outer sole having a specific gravity of 1.16 and a durometer of 60 ± 5 Shore A, with an abrasion factor of 110 or less (DIN 53516). The inner layer has a durometer of 40 ± 5 Shore A. The boots weigh 1,650 grams (a pair, European size 42, including a replaceable polyurethane insole weighing 130 grams). Before beginning training, we measured all the recruit’s shoe heel outsole thickness with a calliper at three positions: medial, posterior and lateral. Soldiers were questioned as to when they began wearing their shoes. At the end of 14 weeks training, the soldiers were questioned regarding having replaced their shoes, and their shoes were re-examined. Those that had replaced their shoes less than 7 weeks previously were requested to bring their old shoes, and if not possible, their data was not used (making the minimum wear time seven weeks). To measure the abrasion, we made transparencies of clocks of different sizes and chose the most appropriate to the size of the heel (Figure 3). With 12 o’clock at the posterior of the heel, the measurements were determined in hours (both sides using the same clock, so three is lateral on the right and medial on the left). We measured the range of the abrasion (e.g. right shoe, from 12 to 4 o’clock) and at the point of maximal abrasion, its hour, the remaining heel thickness, and the width of the abrasion from the periphery towards the centre of the heel. Calculations for the volume of abrasion were based on calculations for a cylindrical wedge [11,12], and then corrected for the ellipsoid shape of the abrasion. To check the assumptions, we compared the measured abrasion width with that calculated from the angles of the span of abrasion, and found them to be reasonable (right and left pooled, measured 30 ± 7 mm, calculated 24 ± 9 mm, *r*^2^ = 0.27, *p* < 0.0001). The volume of abrasion was calculated as the difference between the post-training and pre-training volume calculations.

### Medical follow-up

The soldiers were screened for signs and symptoms of overuse injuries by an orthopaedic surgeon every 2–3 weeks during their training. Diagnosis of stress fractures was based on the clinical examination, X-ray and bone scans [13]. Ankle sprains were graded 1–3 [14]. Patellofemoral pain was diagnosed clinically [15] with specific attention paid to myofascial pain from the muscles of the quadriceps. Achilles tendinitis was diagnosed clinically when signs and symptoms were present in the region of the Achilles tendon 2 to 6 cm proximal to the Achilles bone insertion [16].

### Statistical analysis

Univariate analysis was performed to assess whether any of the baseline measured variables could explain the shoe abrasion. Normal distribution was verified by observing the histograms and with the Kolmogorov-Smirnov goodness of fit test. We compared the baseline and abrasion parameters with Pearson’s correlation and performed Student’s T test for overuse injuries both with baseline and abrasion data. Relevant interval parameters were also ranked and tested with the Chi square test. Overuse injuries in each extremity were analyzed for data on that extremity. All statistical analysis was performed with SAS 9.2.

## Results

There were 76 recruits in the original group, median age 19.2 [interquartile range (IQR): 18.6 to 19.8] years. Baseline parameters are summarized in Tables 1 and 2.

**Table 1:** Baseline parameters

**Parameter**	**Units**	**N**	**Mean** ± **STD**	**Range**
Height	cm	76	177 ± 5	167 to 191
Weight	kg	76	70 ± 7	55 to 88
Tibial length	cm	74	39.7 ± 2.4	34 to 50
Body moment of inertia	kg·m^2^	76	222 ± 32	158 to 298
Right arch height with gauge	mm	74	15 ± 3	3 to 28
Left arch height with gauge	mm	74	14 ± 4	0 to 29
Right arch index		66	0.36 ± 0.08	0.20 to 0.53
Left arch index		66	0.37 ± 0.07	0.23 to 0.52
Right modified arch index		66	0.27 ± 0.27	0.05 to 2.26
Left modified arch index		66	0.23 ± 0.01	0.04 to 0.51
Right foot progression angle	Degrees to lateral	66	7.9 ± 3.5	0 to 16
Left foot progression angle	Degrees to lateral	66	7.6 ± 3.9	0 to 21
Shoe size, European	EU units	71	43.3 ± 1.5	40 to 47

**Table 2:** **Hindfoot angles in degrees** (**mean** ± **SD and range**) **for 58 subjects**

	**Right**		**Left**	
	**Mean** ± **SD**	**Range**	**Mean** ± **SD**	**Range**
Calcaneus varus, standing	−0.8 ± 5.6	−18.0 to 8.7	1.7 ± 4.7	−12.0 to 10.0
Calcaneus varus, tiptoeing	5.2 ± 6.2	−10.0 to 22.7	9.5 ± 7.6	−5.8 to 29.0
Calcaneus-tibial angle, standing	−5.1 ± 7.0	−22.7 to 11.6	−3.2 ± 5.0	−16.0 to 8.5
Heel supination	8.7 ± 5.3	−12.6 to 20.6	9.7 ± 8.1	−9.2 to 34.4

During 14 weeks of basic training, 15 out of 76 (20%) of the recruits were diagnosed with 16 stress fractures (4 femoral, 10 tibial, and 2 metatarsal, one recruit having more than one fracture). Twenty percent of the recruits (15 out of 76) had anterior knee pain, 7 out of 76 (9%) sprained their ankle, and one recruit suffered from Achilles tendinitis.

**Figure 4:**
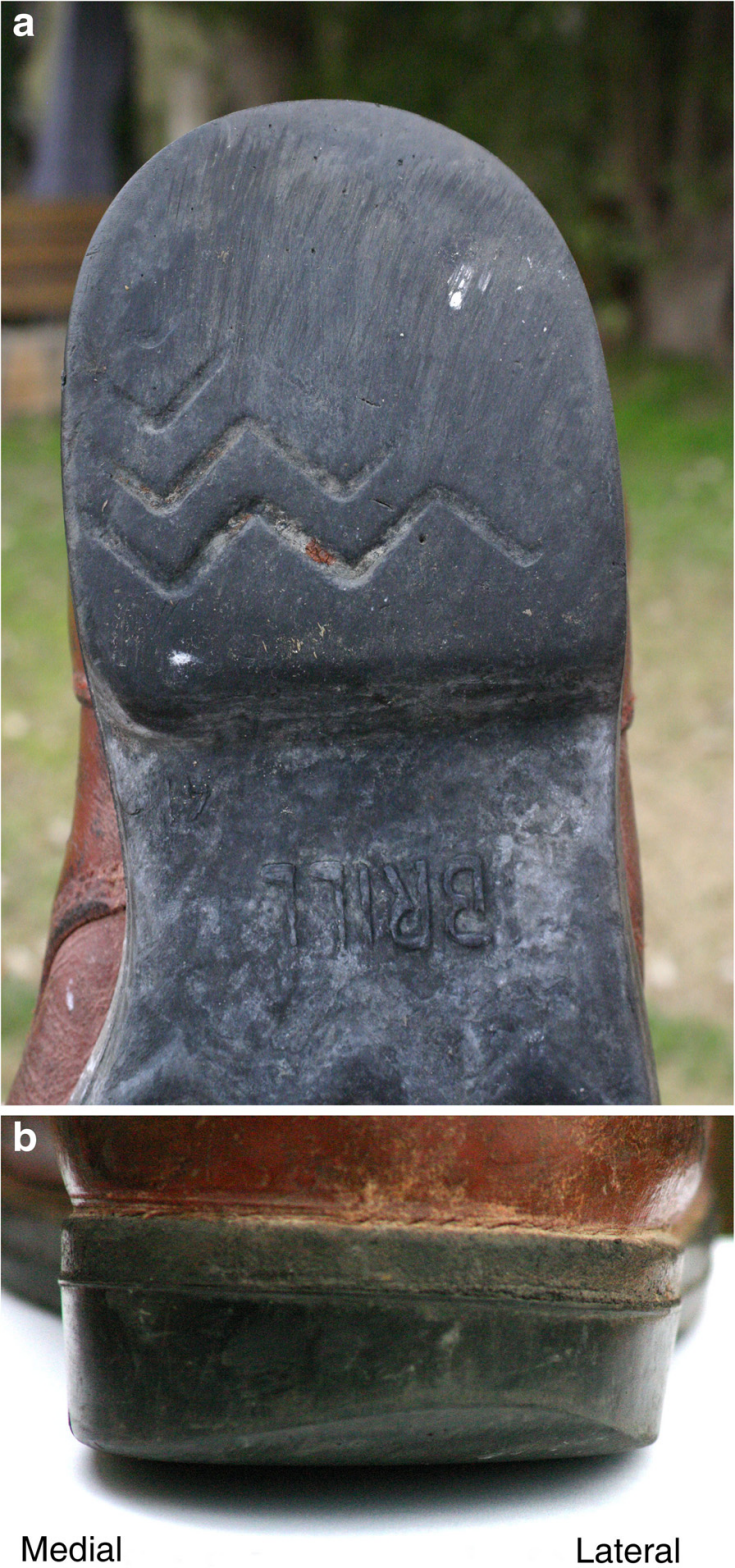
**Shoe heel abrasion.** (**a**) This right shoe is worn from 7 o’ clock to 6 o’ clock (almost all the way round). The maximum abrasion is at 1:30 (laterally, as this is a right shoe). The massive wear can be noted by the disappearance of most of the grooves, also more laterally. (**b**) This causes the shoe to stand slightly slanted to the right (inversion).

Outsole abrasion was 12 (±19 SD) degrees posterolateral on each shoe (Figures 4a and b). The volume of the heel that was eroded was 4.9 (±2.6) cm^3^. Other parameters potentially related to the abrasion or to overuse injuries are presented in Table 3.

**Table 3:** Shoe outsole abrasion data

**Parameter**	**Units**	**N**	**Mean** ± **STD**	**Range**
Max. abrasion direction, right	Degrees to lateral	63	12 ± 16	−30 to 45
Max. abrasion direction, left	Degrees to lateral	63	12 ± 23	−120 to 88
Lateral range of abrasion right	Degrees to lateral	63	73 ± 23	0 to 120
Medial range of abrasion right	Degrees to lateral	63	−59 ± 20	−90 to 60
Lateral range of abrasion left	Degrees to lateral	63	74 ± 15	45 to 105
Medial range of abrasion left	Degrees to lateral	63	−53 ± 21	−90 to 30
Mid range of abrasion right	Degrees to lateral	63	7 ± 16	−30 to 90
Mid range of abrasion left	Degrees to lateral	63	10 ± 11	−8 to 45
Volume of abrasion right	cm^3^	60	5.1 ± 2.8	0.4 to 13.0
Volume of abrasion left	cm^3^	60	4.7 ± 2.5	0.2 to 14.6
Percent of heel abraded right		60	2.7 ± 1.5	0 to 7
Percent of heel abraded left		60	2.5 ± 1.3	0 to 7

On univariate analysis, the angle of maximum wear was related with right foot progression angle (*r* = 0.27, *p* = 0.02), but this finding was not reproduced on the left. Recruits with lateral ankle sprains had higher angles of maximal abrasion (17° ± 14° in the ankle sprain group vs. 10° ± 17° in the group without sprains, *p* = 0.26) and recruits with abrasion on the lateral heel had more lateral ankle sprains [6 out of 44 (14%) vs. 1 out of 32 (3%), *p* = 0.12], but we were obviously underpowered for these calculations. No other abrasion parameter we measured was related to any other baseline measurements (anthropemtric, arch height or hindfoot angles). Body moment of inertia (weight * height^2^) was not found to be related to ankle sprains [14], nor were any other baseline parameters.

Arch height measurements with the 10 mm gauge, and from the foot print plate, were correlated between themselves, but *r* values were not high (gauge - Brosh and Arcan’s alpha *r* = −0.47, *p* < 0.0001, gauge - Chu et al’s arch index, *r* = −0.29, *r* = 0.001, Chu’s modified arch index- Brosh and Arcan’s kappa, *r* = −0.55, *p* < 0.0001).

Recruits with any stress fractures had a marginally higher kappa (heel sharpness factor [9], *p* = 0.05), a lower arch index [8] (*p* = 0.02) and a considerably lower modified arch index (0.20 vs. 0.27, *p* < 0.005), a finding compatible with higher arches. Similar relationships were seen for the femoral and tibial stress fractures, but not metatarsal. Recruits with tibial stress fractures also had shorter tibiae (39.0 vs. 39.8 cm, *p* = 0.05).

## Discussion

According to the literature, there are two main types of shoe heel deformation [17]. Patients with calcaneo-varus in supination tend to flatten out the lateral shoe counter over the heel, also squeezing the lateral aspect of the heel. A second and opposite type has been described in children with over-pronation, which causes medial flattening of the shoe counter sometimes followed by flattening of the medial heel.

In this study, we quantified a third type of abrasion, mainly abrasion of the lateral heel. Most clinicians in the field have observed that most people wear their heels more on the lateral side, as originally noted (to the best of our knowledge) by Barnett et al. [1] in 1956. They attributed this to the initial heel strike on the posterolateral aspect of the heel. In the pathological cases that they described, excessive lateral outsole abrasion was attributed to a pathological “out-turned” foot resulting from a sciatic injury. Other reports on shoe abrasion in the medical literature are mainly descriptive [18,19]. There is some interest in the forensic medicine literature since coroners often try to identify subjects according to their shoes or footprint [20,21].

In spite of the paucity of validated information, patients often receive definitive explanations from the internet about shoe outsole abrasion. Commonly quoted explanations for shoe outsole abrasion patterns are “over-pronation” and “under-pronation”, and these are often translated into recommendations, such as: “Outer sole wear: You turn out. Orthotics may help….Wear on the inner sole: You pronate or turn in. Inner liners or orthotic supports may help” [5].

In the recent medical literature, Barton et al. [18], quotes Vernon et al. [22]: “…greater lateral than medial wear at the heel and forefoot, which may indicate excessive supination”. Vernon et al’s paper is on a Delphi study on podiatrist opinions (level V evidence). They in fact concluded that there is no “one condition, one wear pattern” relationship, refuting previously accepted dogma [22].

Our study is on infantry basic training recruits, probably the healthiest population in Israel. Ninety percent of the recruits continued to train at a greater intensity after the study period, carrying equipment weighing up to 40% of their body weight over tens of kilometers a week. In these healthy subjects, we found great variation in all the parameters we measured. We therefore assume our biomechanical findings to be, at most, normal variation. Our main findings are that there is great variability in how subjects wear down their shoe heels and that shoes wear down more laterally than medially. On average, these recruits eroded nearly 5 cm^3^ of their heel in 14 weeks. The only biomechanical factor found to be related to lateral abrasion is the foot progression angle, a factor clearly suspect, as the lateral heel becomes more posterior the higher the foot progression angle. This is just as predicted by Barnett et al. [1] 54 years ago. We did not find any of the biomechanical factors we measured that are associated with pro-supination or arch height to be related to any outsole abrasion pattern.

In this study we found that recruits with more lateral outsole wear had more lateral ankle sprains. This makes sense, as a laterally worn heel is likely to increase inversion and might elevate the risk for lateral sprains. This concept is supported by an old, but not sufficiently documented method of treatment of recurrent ankle sprains using a wedge under the heel lifting the lateral side. A similar concept that has been reproduced is using a “worm” inside the shoe [23]. While our study design does not enable us to conclude that laterally worn heels cause sprains, it might be reasonable to recommend replacing over-worn shoes, particularly if the wear is lateral. However, we cannot disprove an often quoted statement that the trainee often wears the shoe down so that it is most appropriate for his walking pattern.

In these recruits, we found the previously described intrinsic risk factors for stress fractures of the long bones: short tibiae [24], and low arch [9,25], as measured both by Brosh and Arcan’s heel sharpness factor [9] and Chu’s lower arch index [8]. Metatarsal stress fractures behaved differently, also as previously described [25,26]. These data show that even though this group was smaller than in previous studies, the methods were powerful enough for finding major effects.

This study has several limitations, one being our crude methods of measuring various parameters including subtalar motion, arch height and foot progression angle, and the limited number of variables related to walking pattern. They were dictated by technical limitations where the examinations were performed, but we decided to use these invalidated methods rather than none at all. Another problem is inherent inaccuracy in skin marking techniques. Our results are not considerably different from previous work published on video studies of subjects walking on a treadmill [10,27]. Our standing tibio-calcaneal angle was 4.1° compared with McPoil and Cornwall’s [27] 3.6° and Hetsroni et al’s [10] 4.9°. Our 8.9° range of motion compares with Hetsroni et al’s 7.8°, particularly when taking into account that they measured from heel strike to full pronation only. We also might have measured tibial varum, but as knee alignment abnormalities were exclusion criteria for these recruits, this might have confounded any conclusions.

## Conclusion

Shoe outsole heel abrasion in a training population is a common phenomenon with great variability. Its direction is posterolateral and this is probably related to out-toeing. Ankle sprains may be related to the magnitude of the abrasion. This preliminary study clearly demonstrates the phenomenon in trainees. The abrasion could not be related to subtalar joint motion parameters, so these findings do not warrant modification of subtalar joint motion in order to limit shoe heel abrasion. Additional study is needed to understand the etiology of those cases in which abrasion is excessive.

## Competing interests

The authors declare that they have no competing interests and that there are no sources of funding.

## Authors’ contributions

ASF conceived the study, participated in its design, contributed to the acquisition of data and its analysis and interpretation, and was involved in drafting the manuscript. KP conceived the study, contributed to the acquisition of data and its analysis and interpretation, and was involved in drafting the manuscript. GA conceived the study and participated in its design, and was involved in revising the manuscript critically for important intellectual content. AH contributed to the acquisition of data and its analysis and interpretation, and was involved in revising the manuscript critically for important intellectual content. ET participated in designing the study and was involved in revising the manuscript critically for important intellectual content. CM conceived the study and participated in its design, contributed to the acquisition of data and its analysis and interpretation, and was involved in drafting the manuscript. All authors have read and approved the final manuscript and take responsibility for the appropriate portions of the content.
